# Legacies of Past Exploitation and Climate affect Mammalian Sexes Differently on the Roof of the World - The Case of Wild Yaks

**DOI:** 10.1038/srep08676

**Published:** 2015-03-02

**Authors:** Joel Berger, George B. Schaller, Ellen Cheng, Aili Kang, Michael Krebs, Lishu Li, Mark Hebblewhite

**Affiliations:** 1 Division of Biological Sciences, University of Montana, Missoula, MT 59812, USA; 2 Wildlife Conservation Society, Bronx, NY 10460, USA; 3 Panthera, 8 West 40th Street, New York, NY 10018, USA; 4 Ugyen Wangchuck Institute for Conservation and Environment, Lamai Goempa, Bumthang, Bhutan; 5 China Program, Wildlife Conservation Society, Beijing, China 10024; 6 Rm. 1229, Blvd. 5, Wuliqiao 2nd Street, 1 SectionChaoyang District, Beijing, China 10002; 7 Department of Ecosystem and Conservation Sciences, University of Montana, Missoula, MT 59812, USA

## Abstract

In polar environments, a lack of empirical knowledge about biodiversity prompts reliance on species distribution models to predict future change, yet these ignore the role of biotic interactions including the role of long past human exploitation. To explore how mammals of extreme elevation respond to glacial recession and past harvest, we combined our fieldwork with remote sensing and used analyses of ~60 expeditions from 1850–1925 to represent baseline conditions for wildlife before heavy exploitation on the Tibetan Plateau. Focusing on endangered wild yaks (*Bos mutus*), we document female changes in habitat use across time whereupon they increasingly relied on steeper post-glacial terrain, and currently have a 20x greater dependence on winter snow patches than males. Our twin findings—that the sexes of a cold-adapted species respond differently to modern climate forcing and long-past exploitation—indicate that effective conservation planning will require knowledge of the interplay between past and future if we will assure persistence of the region's biodiversity.

The Himalayan and Tibetan Plateau expanse with some 45,000 glaciers contains the greatest amount of ice and snow outside the poles, and its associated hydrological systems provide sustenance for ~1.5 billion people downstream^1^,^2^. Despite broad global interest in social and economic consequences of rapid warming and glacial recession^3^, little attention has focused on how climate change will affect the region's extreme altitudinal biodiversity^4^. This is puzzling because endemic cold-adapted species are numerous, recognized as cultural icons, play important ecological roles^5^, and can be models for parallel climate challenges in the Arctic^6^,^7^. While similar levels of warming occur simultaneously atop the world and its roof^8^, the mechanisms by which species may, if at all, adjust are not well chronicled^9^.

As early as 1905, Sven Hedin noted “herds of wild yaks …. all of them just at the lower margins of the glaciers” during travels across the Tibetan Plateau^10^. During this gilded exploration period from ~1850–1925 ~60 accounts of high elevation wildlife associated with ice or snow were reported, and these offer a baseline to gauge the extent to which habitat use may have changed across time. In some species for example, past poaching has not only decimated populations regionally (i.e., African elephants, North American bison), but has created a level of historical influences on migration and other behaviours that may persist for decades*—*a phenomenon we heretofore refer to as legacy effects and operationally define as those behavioural attributes of a population shaped by biotic interactions over time (Table 1). Where legacy effects modulate changes, our understanding of ecological responses to environmental conditions will be incomplete unless accounting for both patterns forced by climate and putatively by humans. Approaches such as species distribution or habitat modeling that ignore biotic interactions are often poor predictors of future responses^11^. If one sex is more vulnerable than the other, evidence of 1) differential response to limited resources and/or 2) changes across time in habitat use, should be evident. If support for either exists, then it will be important that conservation planners consider both nuanced and more overt change. Here, we assess synergies between climate-mediated hydrological regimes and human-mediated legacy effects on habitat use in a high elevation extremist, the endangered wild yak^12^, which serves as a totem for the Tibetan Plateau.

## Results

### Analytical Protocols

**Figure 1:**
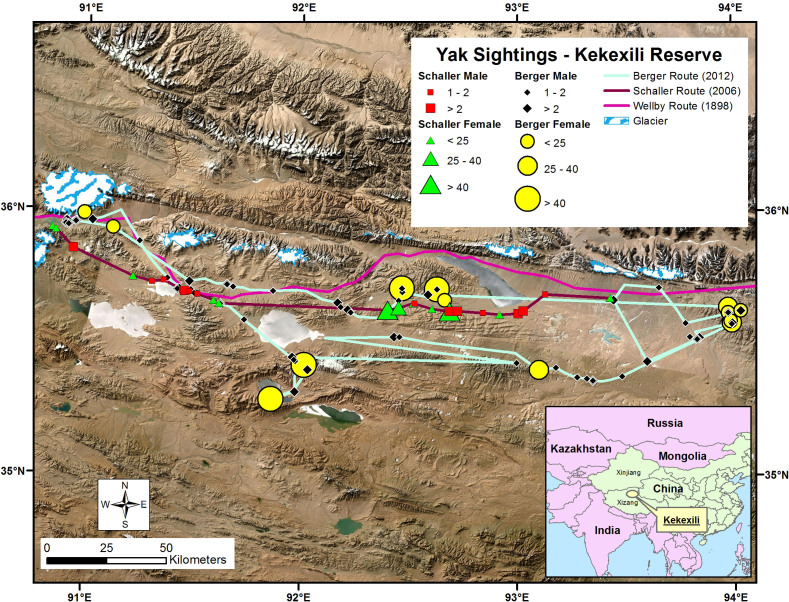
Distribution of yaks by group size and sex in relation to 22 glaciers in western China. Welby's 1896 travel and 2006 and 2012 survey routes shown, the latter two from which Resource Selection was estimated by covariates in Table 2. The map is modified from Berger^15^ and with permission from Allen Press and first published by Oxford University Press, and created from ArcGIS Desktop (version 10.0), with spatial metrics projected to WGS 1984 (UTM) Zone 46 North81. Landsat Global Land Survey (GLS) Shaded Basemaps and natural color, 15-m resolution pan-sharpened Landsat images, enhanced with topographic hill-shading and color balancing with U.S. Geological Survey and the National Aeronautics and Space Administration Landsat images (http://imagery.arcgisonline.com/arcgis/rest/services/LandsatGLS/LandsatShadedBasemap/ImageServer) were used for mapping purposes.

**Figure 2:**
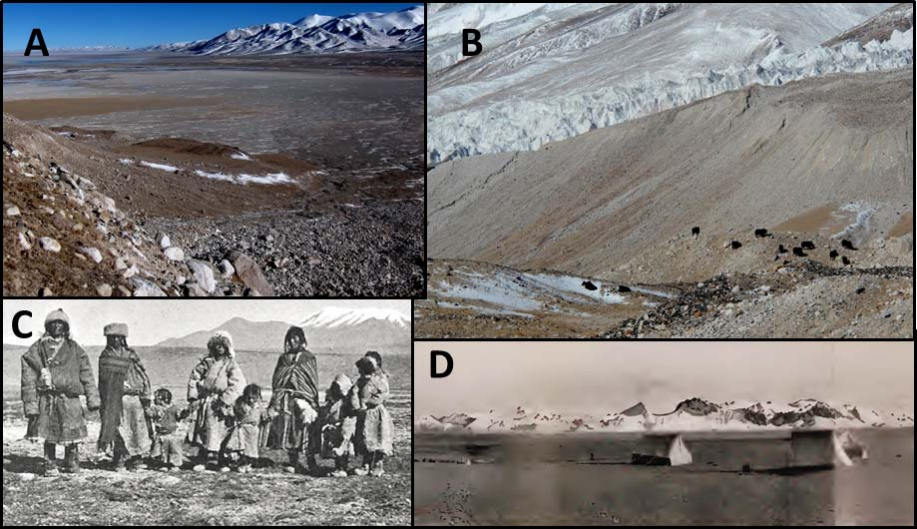
(A) Simple topography from 4,500–5,500 m typical of Tibetan Plateau habitats illustrating steppes (plains and valleys) and sloping hills into mountains and glaciers. (B) Female yak herd in peri-glacial zone, with some resting in snow patch. (C) Tibetan extended family (from Rawlins; SI^22^ and (D) topographies in “A” showing steppe habitat as reported in SI (photo from Hedin^37^). (Fig. 2A and B photographs by J. B.).

We evaluated whether peri-glacial zones represent differentially important habitats to yak, by testing two predictions - that overall yak densities will be greater in and around these high elevation locales, and that females, due to their immediate investment in offspring, should select resources of greater moisture and nutritional quality^13^,^14^,^15^,^16^. Wild yaks are an IUCN Red Data Book listed species restricted to the Tibetan Plateau and obligate grazers of extreme high elevation^5^,^12^. As in other dimorphic ungulates^13^,^14^, the sexes segregate most of the year with females in larger groups than males^15^. We categorized spatial variables using remote sensing of snow, glacial coverage, and vegetation and with field measures, and analyzed these to test our prediction in areas we and an 1896 British military exploration^17^ surveyed more than a century apart; these spanned 1507 km^2^ in 2006^18^ and 4801 km^2^ in 2012 in the 45,000 km^2^ Kekexili National Nature Reserve on the Tibetan-Qinghai Plateau (Fig. 1). Data on sex-specific 2-dimensional habitat use were extricated from 59 19^th^ and 20^th^ century expeditions (Fig. 2, either as 1) on hills or slopes or 2) valleys or steppes) prior to human exploitation (67 male and 81 female groups) and contrasted with modern periods after protection (2006–2012)(137 male and 28 female groups). Poaching intensified between the 1930s and later.

### Peri-glacial Determinants of Habitat Use

**Figure 3:**
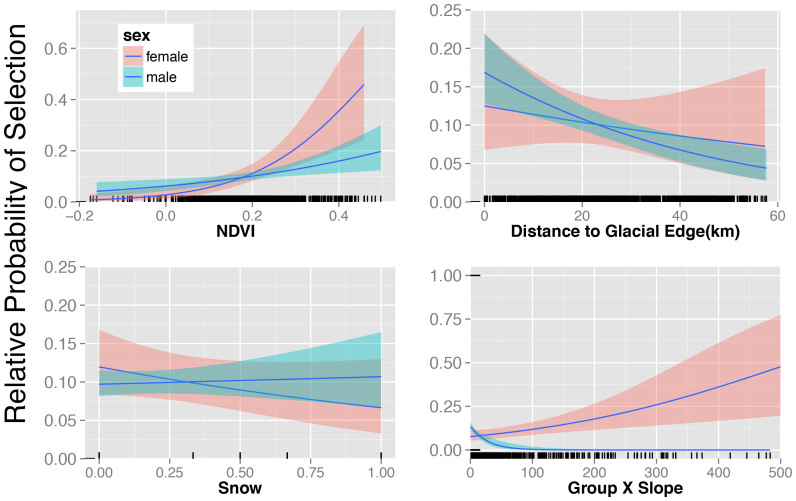
Resource selection by wild yaks for NDVI, snow cover, distance to nearest glacier and the interaction between slope and group size in the Tibetan Plateau during surveys in 2006 and 2012, showing, where supported, group-size (sex) dependent differences in resource selection.

We obtained corresponding resource (habitat) covariates from remote sensing and global datasets (NDVI, snow cover, topography)^15^,^19^ for each yak location during our 2006 and 2012 fieldwork, and tested whether yak group proximity was associated with forage production and distance-to-glacier for all 22 glaciers (Fig. 1, edge and centroid^19^,^20^. Our top Resource Selection Function (RSF) model revealed yak selection for areas closer to glaciers, of higher NDVI, lower snow cover, and shallower slopes (Table 2; Fig. 3). Little uncertainty existed in model selection, with strong support for group size-sex interactive effects; the top model contained 0.82 of the cumulative AIC weight. Inclusion of a random intercept for survey year did not improve model fit.

Mean group size did not differ between survey years (F_1,144_ = 0.40, *P* = 0.52), or through the interaction of sex and surveys (F_1,244_ = 0.69, *P* = 0.40). Only sex had a strong and consistent effect on group size (F_1,144_ = 51.45, *P* < 0.0005) with females in larger groups (32 ± 41.8) than males (2.5 ± 2.03; *P* < 0.001). Because sex and group size were functionally equivalent due to strong differences in group size, we consider sex and group size-based differences equivalent. Effects of sex on resource selection for NDVI, snow and slope were strong with both sexes selecting areas of higher summer growing season NDVI during early winter; females showed greater selection than males (Fig. 3; Table 2). Females also selected steeper slopes (Fig. 3; Table 2); both sexes were nearer to glaciers than expected by chance (Table 2) with females averaging about 30% closer than males (22 *vs* 32 km, respectively). Snow cover affected the overall distribution of yak males and females similarly when explored by satellite imagery, but not when examined at the ground levels (see below).

### Enhanced Female Sensitivity to Snow Patches

**Figure 4:**
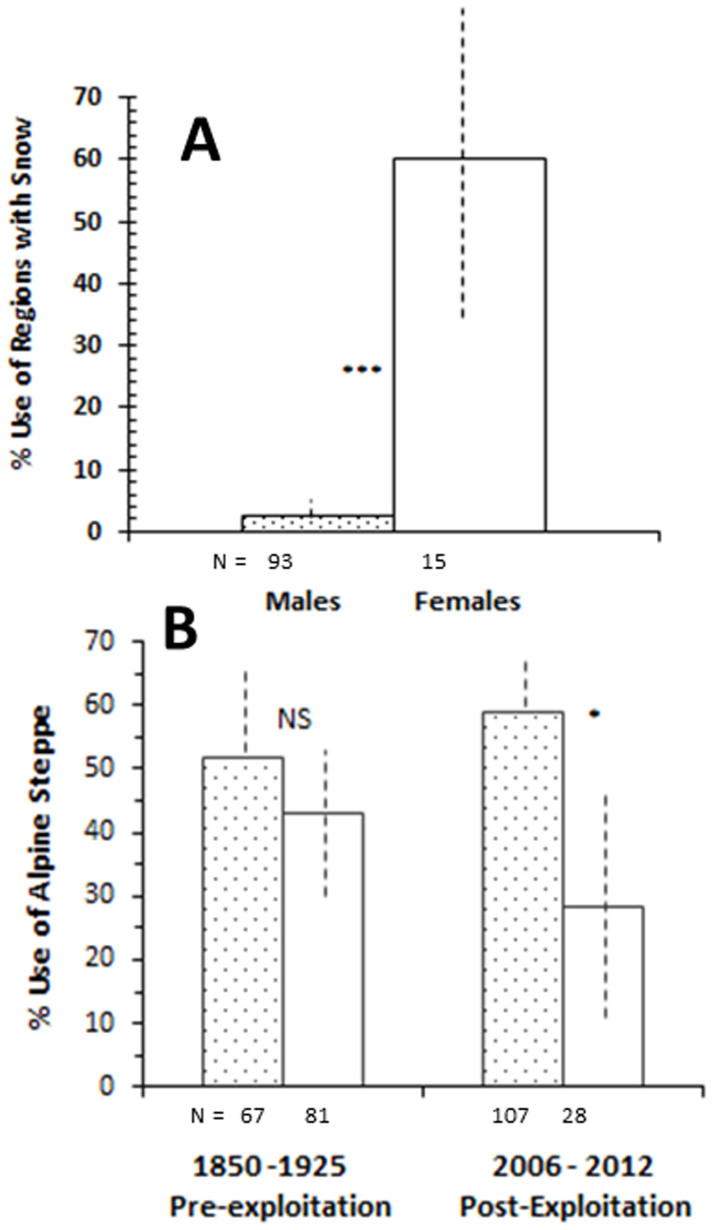
(A) Percent usage of areas within 200 m of snow patches by male and female yak groups in 2012 (*P* < 0.0001). (Odds ratio = 46.50; z = 4.869; for females 95% CI = 35.21% to 84.79% (±24.79); Males 95% CI = −0.37% to 6.81% (±3.59). (B) Temporal changes in female and male yak groups. Baseline (pre-exploitation) period generated from 148 historical and post-exploitation data from recent fieldwork in 2006 and 2012. Sex differences in pre-exploitation period NS, but *P = 0.004 during post-exploitation.

Given that snow was negatively related to resource selection in both sexes (Table 2), our finding – that females were more than 20 times as likely to be within 200 m of snow patches than males (Fig. 4) – was strikingly different than expected. The results of our ground investigations cast doubt on the remote sensing interpretation of aversion to snow (Table 2) and raise the possibility it as an artefact of scale or a lack of understanding of yak ecology, especially because evidence elsewhere demonstrates that, in the absence of snow, moisture in polar environs is unavailable during winter because water is frozen solid^21^.

### Legacy Effects of Past Exploitation

While we report strong effects of peri-glacial zonation on current resource selection in yaks (Table 2, Fig. 3), other factors drive site specificity in habitat use. In social and asocial mammals intense predation has led to striking decadal-long changes in behaviour including habitat abandonment in elephants and moose amongst others (Table 1). If yaks modified patterns of habitat usage as a consequence of past heavy harvest by humans – perhaps by using slopes as a refugium – then, with other factors equal, patterns should vary before and after periods of heavy poaching. To test for a possible legacy effect, we contrasted slope use (Fig. 2) by sex during the ~1850–1925 period with that after protection was established using our recent 2006–2012 post-harvest fieldwork. Prior to weapons more advanced than muzzle-loaders, the sexes used both habitat categories similarly; following strong persecution however females shifted to areas of steeper inclines (Fig. 4), suggesting a greater sensitivity in females. Because most explorations of the Tibetan Plateau (SI) were, like ours, conducted in cold periods when winter travel was doable, the possibility of seasonal differences in habitat selection is not a likely explanation for the decadal differences we report.

Several other possibilities exist. Killing by humans may have created a more intense form of selection on females than males, a possibility which we cannot refute. Nevertheless, given that females occur in larger groups than males and in many mammals larger groups are more difficult to approach^16^, this possibility seems less tenable. Additionally, recent land use by herders or domestic stock might have prompted the changes by females to slopes but this too appears untenable since neither pastoralists nor towns are in the 45,000 km^2^ reserve and livestock are prohibited. It is however possible that male yaks similarly altered habitat use temporarily but they have now rebounded, and currently use steppes as they did about a century ago. Nevertheless, our finding of shifts among females supports the idea of a legacy effect of past exploitation operating more strongly on females than males.

## Discussion

Our results point to both peri-glacial and past human effects on yak behaviour and ecology. Still unexplained is why female show proclivity to snow patches (Fig. 4B). Clearly, the differences between the resource selection models (with its input derived from remote sensing data) and our ground observations of sex differences are in conflict. While both sexes avoided snow, a result that would arise if snow cover is deep, such a finding fails to explain sex differences in proximity to mere snow patches. Two possibilities exist. First, males reduce moisture needs during winter, a hypothesis that remains untested. Second, female nearness to snow, like areas of higher NDVI (Table 2, Figs. 3, 4), may arise as an inevitable consequence of maternal investment. Unlike ungulates from warmer climes, female yaks still lactate in winter^15^. While milk production is physiologically costly^22^ and protein needs are partially adjustable in Arctic ungulates^23^, for yak females, off-sets were ecologically modulated through micro-scale shifts to access 1) moisture that was not frozen solid (hence snow) and 2) vegetation of increased nutritional value (Figs. 3, Table 2). Indeed, winter travelers to the Tibetan Plateau reported abject scarcity of wildlife when snow was absent and rivers and lakes frozen (Supplemental Information), as available water was therefore unavailable.

The distributional ecology of many large mammals has operated through dualities of climatic influence and human persecution, relationships formally noted for at least 150 years^24^,^25^. Our findings of a legacy effect operating on yak females coupled by heightened sensitivity to variation in snow and vegetation suggest potential different outcomes for the sexes as glacial recession continues. Ultimately, given the key role of females in mammalian population dynamics^13^, our results suggest yak populations are likely to be increasingly regulated by changing weather patterns. Population densities may decrease as inferred by the negative relationship to glacial edge (Table 2) and, as documented, across the Tibetan Plateau's west-to-east precipitation gradient^5^. Whether other high elevation specialists or Arctic large mammals employ similar mechanisms to mediate changing conditions awaits additional study.

The Tibetan-Himalayan region's twin traits of aridity and high elevation suggest that with additional warming and more variable precipitation there will be heightened disruptions to key hydrological and ecological processes^1^,^2^,^3^,^26^. Among these is the likelihood of exacerbated conflict between people and wildlife since Tibet's human populations are almost totally dependent upon the same forage for their livestock as that used by endangered wild yaks and kiang^27^,^28^,^29^. Enhanced knowledge about how native wildlife will respond to environmental change and past exploitation as evidenced by legacy effects will facilitate crafting prudent solutions that benefit human livelihoods as well as associated high elevation biodiversity.

## Methods

### Study Area and Assessment of Legacy Effects

We employed drive transects in the Kekexili region during winter in 2006 and 2012, classifying yak groups by age and sex using 3-km survey buffer zones (Fig. 1). We also extricated data from 59 19^th^ and 20^th^ century explorers' accounts (Supplementary Information; SI) from which we obtained 148 locations by habitat for the period 1850–1925. We then used our 2006–2012 data gathered during early winter to contrast periods before and after periods of human persecution in the mid-to-late 20th century^5^,^12^. The temporal contrasts enabled appraisal of potential changes in yak habitat use as a result of heavy exploitation.

To explore the potential for legacy effects, we checked aforementioned historic travel accounts in the Tibetan-Himalayan region, under the assumption that in the absence of modern weapons and organized shooting, species would be more abundant and less harassed than after. While the detection of legacy effects will be enhanced best through longitudinal analyses across multiple generations and comparative study, migrations, habitat shifts, activity patterns and personality change when influenced by heavy predation even where longitudinal data are lacking (Table 1). Based on the historical analyses (SI) we assigned 148 observations to groups of males or females by the same habitat categories that we recorded in our current (2006, 2012) surveys.

### Remote Sensing of Topography, Glaciers, Snow, and NDVI

We created digital elevation models (DEMs)^15^, and assessed Normalized Difference Vegetation Index (NDVI), snow, and glaciers using ArcGIS Desktop (version 10.0) to display all 2006 and 2012 yak locations as spatial metrics (WGS 1984 Universal Transverse Mercator (UTM) Zone 46 North)(ESRI; ArcGIS Desktop Help-Cell size and resampling in analysis.)^30^. Glacier delineation involved Landsat imagery (Global Land Survey 2010; http://glovis.usgs.gov) and consisted of individual tiles derived from both Landsat 7 ETM + and Landsat 5 TM satellites collected between 2008 and 2011 (details in SI).

Glacier sizes were determined from hand-digitization using ArcGIS vector construction and editing tools with boundaries between 2000 and 2010 contrasted using Global Land Survey raster datasets beforehand to ensure accuracy of digitized glacial perimeters and to exclude snow or snowfields. With these polygons, total areas and centroids for each glacier were calculated for each 2006 and 2012 yak location and distance to glacial metrics evaluated using model selection procedures (SI). MODIS (Moderate Resolution Imaging Spectroradiometer) data were used to appraise NDVI and snow extent cover. Both NDVI and maximum snow cover datasets consist of 16-day 250 m resolution and 8-day 500 m resolution, respectively, for data periods and yak locations in both 2006 and 2012 (SI). NDVI is associated with forage biomass^31^,^32^ and quality for large herbivores^33^,^34^. Mean NDVI was computed using three datasets spanning late July and August to estimate peak vegetation for each observation year; snow cover was estimated in October, November, and December (SI). We expressed snow cover via MODIS as the percent of time covered during the period of yak observations (SI).

On the ground, we assessed yak proximity to snow patches in 2012 either by laser rangefinder or by projection of distances between the centroid of a herd to the nearest patch. Projections were based on number of approximated yak body lengths using a standard of 2.5 m. We estimated the proportional snow coverage of areas radiating from each group's ~ centroid using step-toe transects which covered 0.04 km^2^ per group. Snow coverage averaged only 4.42% (±4.036 SD, N = 8) within the 0.04 km^2^ sampled units yet in all cases females walked to and consumed snow.

To evaluate the probability of resource selection by sex as a function of group size for glacial and primary productivity-related spatial covariates, we used RSF^35^,^36^. We applied mixed-effects models to account for potential differences in yak distribution using a random intercept for survey years.

### Analytical Approaches and Modeling of Resource Selection

Transect routes throughout the study area were created from GPS point locations of all large mammal species observed using vector spatial tools in ArcGIS. These locations were first ordered chronologically and then converted to a single polyline feature containing vertices at each location. They were then split at their vertices into individual line segments and the linear distance between each chronological point described using the Calculate Geometry function.

We assessed potential differences in contemporary yak resource use and selection for glacial and primary productivity-related spatial covariates using RSF^35^ to evaluate roles of sex (*S*) and group size (*Grp)*. To test our hypotheses about differences in resource selectivity we adapted a group-size dependent RSF developed for group-living bison^36^. We estimated the relative probability of yak use (*w(x)*) of *k* spatial covariates (*X*_1_, …, *k*) as well as both the effects of *Grp* and *S* on the selection for independent variables *X*_1,2 _using a sex and group-size dependent RSF^33^, by:
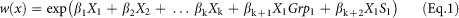
where β_k + 1_ *X*_1_*Grp*_1 _is the selection coefficient representing the effects of *Grp* on the covariate X_1_, and β_k + 2_ *X*_1_*S_1_*is the selection coefficient representing the effects of *S* on selection for the covariate *X*_1_. Because random locations were assigned to a random *Grp* and *S,* drawing from the distribution of observed group sizes and sex frequencies, the consequent group-size and sex-dependent selection coefficients were evaluated through these interactions alone; main effects of *S* and *Grp* have no direct interpretation. Moreover, since *Grp* was uniformly associated with *S* in our wild yak data, we did not include *Grp*-*S* interaction coefficients in the same model. In other words, because associations between sex and group size were virtually overlapped, we evaluated models containing either interactions for the sex- or group size-interaction. Rather, we addressed this problem through model selection by creating an *a-priori* list of models with main effects of the spatial resource covariates (*X*_1_, …, *k*) and contrasting formulations of either *Grp*- or *S*-dependent interactions (SI).

## Author Contributions

J.B., M.H. and E.C. conceived of the analytical framework while J.B., G.B.S., E.C., L.L. and A.K. participated in the field work. G.B.S. began initial study of yaks and other ungulates in the region three decades ago. M.K. did the GIS and remote sensing analyses, and M.H. did the modeling. J.B. with help from G.B.S. accessed the historic literature and J.B. did the analyses.

## Supplementary Material

Supplementary Information Supplementary Information

## Figures and Tables

**Table 1:** Key examples of legacy effects in mammalian behavior or ecology as mediated directly or indirectly by anthropogenically-changed predation regimes including carnivore expansion and reintroduction. *Numbers reflect references in Supplemental Information

Behavior or Ecology	Species	Location	Selective Forces	Effect*
**Migration**	Elephants, elk	East Africa, Rocky Mts, USA	Human hunting	Altered routes^2^,^3^,^4^,^5^
**Distributional shift**	Moose, elk	Yellowstone, USA; Banff, Canada	Bear, wolf predation	Use of humans as predation shields^6^,^7^
**Activity Peaks**	Coyotes	Western USA	Human hunting	Decrease diurnal behavior^8^,^9^
**Personality**	Many mammals	Global	Human hunting	Increased shyness or vigilance^10^,^11^
**Diet Selection**	Black bears, wolves	Yosemite, Yellowstone, USA	Novel foods	‘Pack’-mediated learning^12^,^13^

**Table 2:** Parameter estimates for wild yak resource selection during Nov/Dec, 2006 and 2012, on the Tibetan Plateau. Parameter definitions are as follows: NDVI = normalized difference vegetation index for the previous summer's growing season (July-August); Snow = percentage snow cover occurring during the survey; Slope = degree's slope of the terrain; Distance to Glacier (DG) = distance to nearest glacier edge in km

	Estimate	Std. Error	Z-value	Pr(>IzI)
**Intercept**	−2.300	0.2771	−8.301	2.11E-10***
**NDVI**	7.730	1.2793	6.042	1.52E-09***
**Snow**	0.723	0.3836	1.884	0.0596
**Slope**	−0.197	0.0572	−3.452	0.0006***
**Dist-Glacier (DG)**	−0.040	0.0077	−5.5226	1.74E-07***
DG*NDVI	0.084	0.0568	1.482	0.1383
DG*Snow	−0.051	0.0239	−2.112	0.0347*
DG*Slope	0.007	0.0029	2.305	0.0212*

## References

[b1] Hansen J. *et al.* Black Soot and the survival of Tibetan glaciers. Proc. Nat. Acad. Sci. 106, 22114–22118 (2009). 19996173 10.1073/pnas.0910444106 PMC2790363

[b2] Jianping Y., Yongjian D. & Shiyin L. Variations of snow cover in the source regions of the Yangtze and Yellow Rivers in China between 1960 and 1999. J. Glaciology 53, 420–426 (2007).

[b3] Xu J. *et al.* The melting Himalayas: cascading effects of climate change on water, biodiversity, and livelihoods. Cons. Biol. 23, 520–530 (2009). 10.1111/j.1523-1739.2009.01237.x 22748090

[b4] Post E. *et al.* Ecological dynamics across the Arctic associated with recent climate change. Science 325, 1355–1358 (2009). 19745143 10.1126/science.1173113

[b5] Schaller G. B. Wildlife of the Tibetan Steppe (Univ. Chicago Press, 1998).

[b6] Brodie J., Post E. & Doak D. (Eds.) Climate Change and Wildlife Conservation (Univ. Chicago Press, 2012).

[b7] Post E. *et al.* Ecological consequences of sea-ice decline. Science 341, 519–524 (2013). 23908231 10.1126/science.1235225

[b8] Qiu J. The third pole. Nature 454, 393–396 (2008). 18650887 10.1038/454393a

[b9] Moritz C. *et al.* Impact of a century of climate change on small-mammal communities in Yosemite National Park, USA. Science 322, 261–264 (2008). 18845755 10.1126/science.1163428

[b10] Hedin S. Southern Tibet. Vol III. (Lithograph Institute, Stockholm of the General Staff of the Swedish Army, 1922).

[b11] Post E. *et al.* Global population dynamics and hotspots of response to climate change. BioScience 59, 489–497 (2009).

[b12] Leslie D. M. & Schaller G. B. *Bos grunniens* and *Bos mutus* (Artiodactyla: Bovidae). Mammal. Spec. 836, 1–17 (2008).

[b13] Conradt L., Clutton-Brock T. H. & Guinness F. E. Sex differences in weather sensitivity can cause habitat segregation: red deer as an example. Anim. Beh. 59, 1049–1060 (2000). 10.1006/anbe.2000.1409 10860532

[b14] Bowyer R. T. Sexual segregation in ruminants: definitions, hypotheses, and implications for conservation and management. J. Mamm. 85, 1039–1052 (2004).

[b15] Berger J. *et al.* Sex differences in ecology of wild yaks at high elevation in the Kekexili Reserve, Tibetan-Qinghai Plateau, China. J. Mamm. 95, 638–645 (2014).

[b16] Berger J. Pregnancy incentives, predation constraints, and habitat shifts: Experimental and field evidence for wild bighorn sheep. Anim. Beh. 41, 61–77 (1991).

[b17] Welby M. S. Through Tibet to China. Geograph. J. 12, 262–278 (1898).

[b18] Schaller G. B., Kang A., Hashi T. D. & Cai P. A winter wildlife survey in the northern Qiangtang of Tibet Autonomous Region and Qinghai Province, China. Acta Therio. Sinica 27, 309–316 (2007).

[b19] Huete A. K. *et al.* Overview of the radiometric and biophysical performance of the MODIS vegetation indices. Remote Sens. Environ. 83, 195–213 (2002).

[b20] Hebblewhite M., Merrill E. H. & McDermid G. A multi-scale test of the forage maturation hypothesis for a partially migratory montane elk population. Ecol. Monog. 78, 141–166 (2008).

[b21] Klein D. A. Arctic ungulates at the northern edge of terrestrial life. Rangifer 16, 51–56 (1996).

[b22] Robbins C. T. Wildlife Feeding and Nutrition (Academic Press, San Diego, CA, 1993).

[b23] Parker K. L., Barboza P. S. & Stephenson T. R. Protein conservation in female caribou (*Rangifer tarandus*): Effects of decreasing diet quality during winter. J. Mamm. 86, 610–622 (2005).

[b24] Darwin C. R. On the Origin of Species by Natural Selection (Murray, London, 1859).

[b25] Wallace A. R. Tropical Nature, and Other Essays (Macmillan Co. London, 1878).

[b26] Dahe Q., Shiyin L. & Peiji L. Snow cover distribution, variability, and response to climate change in Western China. J. Climate 19, 1820–1833 (2006).

[b27] Jiang Z. & Xia W. The niches of yaks, Tibetan sheep, and plateau pikas in the alpine meadow ecosystem. Acta Biol. Plat. Sin. 6, 115–146 (1987).

[b28] Sheehy D. P., Miller D. & Johnson D. A. Transformation of traditional livestock systems on the Tibetan steppe. Secheresse 17, 142–151 (2006).

[b29] Harris R. B. Wildlife Conservation in China: Preserving the Habitat of China's Wild West (Sharpe, M. E., Armonk, 2008).

[b30] Burrough P. A. & McDonnell R. A. Principles of Geographical Information Systems (Oxford Univ. Press Inc., 1998).

[b31] Kawamura K. *et al.* Monitoring of forage conditions with MODIS imagery in the Xilingol steppe, Inner Mongolia. Internat. J. Remote Sensing 26, 1423–36 (2005).

[b32] Xu B. *et al.* MODIS-based remote sensing monitoring of grass production in China. Internat. J. Remote Sensing 29, 5313–5327 (2008).

[b33] Mysterud A., Langvatn R., Yoccoz N. G. & Stenseth N. C. Plant phenology, migration and geographical variation in body weight of a large herbivore: the effect of a variable topography. J. Anim. Ecol. 70, 915–923 (2001).

[b34] Pettorelli N. *et al.* Using the satellite-derived NDVI to assess ecological responses to environmental change. Trends Ecol. and Evol. 20, 503–510 (2005). 10.1016/j.tree.2005.05.011 16701427

[b35] Boyce M. S. & McDonald L. L. Relating populations to habitats using resource selection functions. Trends Ecol. Evol. 14, 268–272 (1999). 10370262 10.1016/s0169-5347(99)01593-1

[b36] Fortin D. *et al.* Group-size-mediated habitat selection and group fusion-fission dynamics of bison under predation risk. Ecology 90, 2480–2490 (2009). 19769126 10.1890/08-0345.1

[b37] Hedin S. Trans-Himalaya. Vol 1. (Macmillan, NY, 1910).

