# Supplementary material for: Legacies of Past Exploitation and Climate affect Mammalian Sexes Differently on the Roof of the World - The Case of Wild Yaks
Source: Sci Rep. 2015 Mar 2;5:8676. doi: 10.1038/srep08676 (PMC4345326; doi:10.1038/srep08676)
Supplement: Supplementary Information [file srep08676-s1.pdf]

Additional; - Supplementary Information

The material below is intended to accompany this paper (at http://www.nature.com/ Scientificreports)

Legacies of Past Exploitation and Climate affect Mammalian Sexes Differently on the World's Roof – The Case of Wild Yaks

Authors: Joel Berger[1, 2*], George B. Schaller[3], Ellen Cheng[4], Aili Kang[5], Michael Krebs[1], Lishu Li[6], Mark Hebblewhite[7]

*Correspondence to: jberger@wcs.org

This supplement provides additional details of protocols and methods, historical data, remote sensing, analytical approaches and modeling of resource selection.

Supplementary Figures S1–S2. (S1) Relative data contribution of historical sources. (S2) Relationships between group size and sex and latitude and longitude across the Kekexili study region during 2006 and 2012.

Supplementary Table S1. Model selection results for wild yak resource selection functions from late fall, winter, 2006 and 2012 on the Tibetan Plateau.

Legacy Effects as Inferred from Historical Data. As indicated, legacy effects were operationally defined as those behavioural attributes of a population shaped by biotic interactions over time. While many cases will therefore be evolutionary in nature[1], in our case we rely on decadal or relatively immediate (i.e. proximate) responses reflected by behavioural plasticity especially those associated with extreme exploitation. The detection of legacy effects will be enhanced best by analyses across multiple generations and comparative study. References from Table 1 are as follows: migration[2–5], distributional shifts[6-7], activity[8-9], personality[10-11], and diet selection[12-13]. Other taxa with documented or suspected legacy effects of intense exploitation include cetaceans[14], primates[15-16], and marsupials[17].

To check the possibility of legacy effects in wild yaks, we examined 59 historic expeditions across the Tibetan-Himalayan region to establish baseline values[18-77] of habitat use prior to periods of widespread shooting (which began after the 1930s). The first historical account, written by Bogle in 1774 and made available in 1876[20], was followed by Moorcroft and Trebeck in 1841[21] and included their 1819–1825 journeys to part of Ladakh, Kashmir, and associated regions. The last was Kingdon Ward's (1937) *Across Southern Tibet in 1935,* which includes the crossing of 3 passes >5,500 m and reference to domestic but not wild yak[76]. In most cases explicit mention of yak presence or absence is recorded: "The wild yak is not found anywhere in this region" (Wollaston 1922:7)[77]. Expeditions were taken primarily during winter because summer travel was inefficient due to rivers and sodden areas[18, 19, 22].

Surveyors often reported habitat, groups, or sex. For instance, Prejevalsky (1876), "the southern slope of this range that we saw herds of them" or "I was clambering over the mountains when I suddenly caught sight of three yaks lying down" [33, 50], Welby (1898) "I

spotted in a valley… a single yak grazing"[23]; and Rawlins 1905 "whilst immense herds of wild yak were to be seen grazing in all the valleys"[22].

Twenty-eight expeditions yielded 217 descriptions in which yak habitat, group, or sex were inferable. The word nullah was used in 9 of these, 2 for putative female groups and 7 of males in small groups or singly. Because it was unclear where nullahs were situated and because the sample size for nullah usage itself was insufficient for classification as a habitat, these 9 cases were censored. Likewise, of the total 208 remaining accounts, 60 did not include simultaneous mention of both habitat and sex (or its inference), so these were excluded, rendering 148 usable accounts (Fig. S1).

Of these, 25 surveys contributed 65% of the data. Rawlins (1905)[22], Welby (1898)[23], and Deasy (1901)[26] contributed the remaining ~35%. We included accounts from 6 of Hedin's travels[25, 29, 31, 38, 40, 42] in the usable data set (Fig. S1) but excluded observations from his 1925 book[18] since it summarized much of his prior explorations.

To assess whether groups noted in the historical record were of males or females, we relied on three methods. First, we used direct surveyors' notation and excluded cases in which we could not reasonably make a determination. Second, we based inferences on currently known proclivities of male and female sexually dimorphic ungulates to associate in different-sized groups[78-80]. However, this pattern was noted for wild yaks nearly 125 years ago: "the cows are generally to be found in herds varying in numbers from ten to one hundred, while the old bulls are for the most part solitary or in small parties of three or four" (Kinloch 1892:118)[27], and well before the heavy human exploitation period. The groups we observed fit well within these early-noted size categories for the two sexes. For instance, in 2012 the largest female group was ~210. Median group size for females was 23. For males it was 1, and 2/3rds of those consisted of 1–2

individuals. Mean group size for females [34.27 (95% CI=7.60 to 60.94)] was about 15x larger than that for males [2.41 (95% CI=1.27 to 3.26)](z=-5.83; P<0.001)[80].

Finally, we assumed groups to be of males when 7 or less, an assumption that we previously assessed by contrasting group sizes between those having been documented with known males and those with presumptive males. If differences in these categories of males existed, the assumption would be invalid. However, means and 95% CI of known and putative male groups were similar: 2.56 (1.97 to 3.21) and 2.59 (1.95 to 3.17); (P=0.947; $t_{1,93}$ = 0.066)[80]. All known female groups contained more than 7 individuals. Overall, these three assessments enabled an evaluation of the possibility of a legacy effect.

Procedures Used in Remote Sensing. As indicated, we used ArcGIS Desktop (version 10.0) to display all yak locations with spatial metrics projected to WGS 1984 Universal Transverse Mercator (UTM) Zone 46 North[81] (Fig. 1). Landsat Global Land Survey (GLS) Shaded Basemaps (available as online coverage through the ESRI Image Service) and natural color, 15-m resolution pan-sharpened Landsat images, enhanced with topographic hill-shading and color balancing with U.S. Geological Survey and the National Aeronautics and Space Administration Landsat images (http://imagery.arcgisonline.com/arcgis/services/LandsatGLS/LandsatShadedBasemap/ImageServer) were used for mapping purposes.

These global minimal-cloud cover, ortho-rectified Landsat data products support global assessments of land-cover, land cover-change, and ecosystem dynamics. For each yak group slope usage was determined using Shuttle Radar Topography Mission (https://lta.cr.usgs.gov/SRTM2) images, which are 3 arc-second in resolution (approximately 90

m), collected in 2000 and available in 1-degree tile formats. Metrics of inclines were derived from the Slope function in ArcGIS Spatial Analyst, and steepness was expressed in degrees from horizontal (0–90), and bi-linear interpolation to re-project and re-sample the raster to provide more reliable estimates of individual incline values[81-84]. Output cell sizes for all slope surfaces were at approximately 100 m.

To evaluate the probability of resource selection by sex as a function of group size for glacial and primary productivity-related spatial covariates, we used RSF[85]. We applied mixed-effects models to account for potential differences in yak distribution using a random intercept for survey years.

Analytical Approaches and Modeling of Resource Selection. To test for broad differences in habitat use and to relate it to historical surveys, we first compared frequencies of use between sexes and as a function of group size. We also conducted direct comparison of resources used by males and females, using logistic regression to check the possibility of sex differences. We next evaluated resource selection with a used-available design comparing the covariate values at locations of yaks compared to random locations within a 3-km buffer of the survey route. This approach was conservative because it effectively assumed equal sightability within our survey buffer although yaks – due to their larger size and flat sparsely vegetated survey areas – may be detectable at greater distances[86]. We generated 1 random location for each 1-km of each survey in each year for comparison to actual yak locations.

For all models, we used scatterplot matrices (i. e. Fig. S2) and a threshold correlation coefficient of r=0.5 to screen for collinearity among spatial covariates; model selection was conducted using Akaike Information Criteria[87-89]. In screening for co-linearity amongst spatial

covariates with scatterplot matrixes and correlation coefficient of r=0.5, we noted our two different measures of distance to glaciers, their edge and centroid, were too correlated to include in the same model (r>0.7). Distance to glacier edge explained yak resource selection more than distance to center of glacier, so we retained distance to glacier in subsequent modeling. No other covariates were correlated (r>0.5), though distance to glacier edge and glacier centroid were close (r=0.43). Model selection was conducted using Akaike Information Criteria (AIC) using the number of independently observed yak groups as the sample unit. K-folds cross validation of the top RSF model was performed[87] with all analyses conducted in R[90].

Latitude:longitude plots for the sexes suggested no skew in a given direction (Fig. S2). And, while as the effects of sex on resource selection for NDVI, snow and slope were strong (Fig. 3; Table 2), AIC evidence ratio of averaged group vs sex effects was 9.1 (Table S 1). Thus, we retained group-size effects in the best RSF model, not sex, although both are interchangeable for wild yaks given our sex-group size distributions. Model fit was not improved by inclusion of a random intercept for survey year (i. e. ∆AIC between the top model in Table S1 with and without the random intercept revealed the fixed-effects model in Table S1 had better model fit). The overall model fit was highly significant with a likelihood ratio test X^2=6.43, p<0.00001, and a Hosmer-Lemeshow test statistic (p= 0.11) confirming adequate fit across all predicted values of resource selection. Model classification measured by ROC was satisfactory, with an Area under the Curve (AUC) of 0.778, reflecting moderate to high model classification success. Cross-validation using 5 random partitions revealed the average Spearman rank correlation between predicted and observed frequency of use was r_s=0.77 (SE=0.0085), confirming moderate-high predictive capacity.

Cross-validation results between the 2006 and 2012 survey years were not excessively strong (r=-0.22, SE=0.5), despite the lack of support for a random intercept for survey year, suggesting that the differing snow conditions during the surveys (mean snow cover in 2006 was 0.55; 2012, 0.11) may have affected the predictive performance between years. However, overall, the top yak RSF model correctly classified 76% of yak locations in 2006 and 84% in 2012. Hence, despite expected variability between survey years, our average model captured yak resource selection between years reasonably well.

References

1. Allendorf, F. A, & Hard, J. J. Human-induced evolution caused by unnatural selection through harvest of wild animals. *Proc. Nat. Acad. Sci*. **106**, 9987–9994 (2009).

2. Shannon, G. *et al.,* Effects of social disruption in elephants persist decades after culling. *Front. Zool.* **10**, 62 (2013).

3. McComb, K., Moss, C., Durant, S. M., Baker, L., & Sayialel, S. Matriarchs as repositories of social knowledge in African elephants. *Science* **292**, 491–494 (2001).

4. Gobush, K. S., Mutayoba, B. M., & Wasser, S. K. Long-term impacts of poaching on relatedness, stress physiology and reproductive output of adult female African elephants. *Cons. Biol.* **22**, 1590–1599 (2008).

5. Connor, M. M., White, G. C., & Freddy, D. J. Elk movement in response to early-season hunting in northwest Colorado. *J. Wildl. Manage.* **6**, 926–940 (2001).

6. Hebblewhite. M., *et al.,* Human activity mediates a trophic cascade caused by wolves. *Ecol.* **86**, 2135–2144 (2005).

7. Berger, J. Fear, human shields, and the re-distribution of prey and predators in protected areas. *Biol. Let.* **3**, 620–623 (2007).

8. Kitchen, A. M., Gese, E. M., Schauster, R. E. Changes in coyote activity patterns due to reduced exposure to human persecution. *Can. J. Zool*. **78**, 853–857 (2000).

9. Vila, C., Urios, V., & Castroviejo, J. in *Ecology and Conservation of Wolves in a Changing World*, L.N. Carbyn, S.H. Fritts, D.R. Seip, Eds. (Canadian Circumpolar Institute, Univ. Alberta Press, Edmonton, 1992), pp. 335-340.

10. Caro, T. M., Demography and behaviour of African mammals subject to exploitation. *Biol. Cons*. **91**, 91–97 (1999).

11. Berger, J., *The Better to Eat You With: Fear in the Animal World* (Univ. Chicago Press, 2008).

12. Breck, S. W., Lance, N., & Seher, V. Selective foraging for anthropogenic resources by black bears: Minivans in Yosemite National Park. *J. Mamm*. **90**, 1041–1044 (2009).

13. Smith, D. *et al.*, Wolf–bison interactions in Yellowstone National Park. *J. Mamm.* **81**, 1128–1135 (2000).

14. Wade, P. R., Reeves, R. R., & Mesnick, S. L. Social and behavioural factors in cetacean responses to overexploitation: are odontocetes less "resilient" than mysticetes. *J. Marin. Biol*. doi:10.1155/2012/567276 (2012).

15. Isbell, L. A. Sudden short-term increase in mortality of vervet monkeys (*Cercopithecus aethiops*) due to leopard predation in Amboseli national park, Kenya. *Am. J. Primatol*. **21**, 41–52 (1990).

16. Sapolski, R. A. *A Primate's Memoir: A Neuroscientist's Unconventional Life among the Baboons* (Simon Shuster, NY, 2007).

17. Blumstein, D. A., & Daniel, J. C., The loss of anti-predator behaviour following isolation on islands. *Proc. Royal Soc*. **272**, 1663–1668 (2005).

18. Hedin, S. *Southern Tibet. Vol III.* (Lithograph Institute, Stockholm of the General Staff of the Swedish Army, 1922).

19. Welby, M. S. Through Tibet to China. *Geograph. J.* **12**, 262–278 (1898).

20. Bogle Narratives of the Mission 1876 (1774), in *Narratives of the Mission of George Bogle to Tibet, and of the Journey of Thomas Manning to Lhasa, edited, with notes, and introduction and lives of Mr Bogle and Mr Manning,* C. R. Markham, Ed. (London, 1876, Reprinted in New Delhi, Manjusri Pub. House, 1971).

21. Moorcroft, W. & Trebeck, G. *Travels in the Himalayan Provinces of Hindustan and the Panjab, in Ladakh and Kashmir; in Peshawar, Kabul, Kunduz, and Bokhara, from 1819 to 1825* (John Murray, London, 1841).

22. Rawlings, C. G. *The Great Plateau. Being an Account of Exploration in Central Tibet, 1903, and of the Gartok Expedition, 1904-1905* (Edward Arnold, London, 1905).

23. Welby, M. S. *Through Unknown Tibet* (Fisher, Unwin, London, 1898).

24. Bower, H. *Diary of a Journey across Tibet* (Rivington, Percival and Co., London, 1894).

25. Hedin, S. *My Life as an Explorer* (Garden City Publ., NY, 1925).

26. Deasy, H. H. P. *In Tibet and Chinese Turkestan* (Fisher Unwin., London, 1901).

27. Kinlock, A. *Large Game Shooting in Thibet, the Himalayas, Northern and Central India* (Thacker, Spink, and Company, Calcutta, 1892).

28. Phelps, E. Yak shooting in Tibet. *J. Bombay Natural History Soc.* **13**, 134–143 (1900).

29. Hedin, S. *Adventures in Tibet* (Hurst and Blackett, London, 1904).

30. Rockhill, W. W. *Land of the Lamas: Notes of a Journey through China Mongolia and Tibet* (Century Co., NY, 1891).

31. Hedin, S. *Scientific Results of a Journey in Central Asia, 1899-1902, Vol III. North and East Tibet* (Lithograph Institute, Stockholm, 1905).

32. Sandberg, G. *Exploration of Tibet* (Thacker, Spink & Co., London, 1904).

33. Prejevalsky, N. *Mongolia the Tangut Country and the Solitudes of Northern Tibet. Vol. 1* (Sampson, Low, Marston, Searle, and Rivington, London, 1876).

34. Bonvalot, G. *Across Thibet* (Cassekk & Co., London, 1892).

35. Reid, W. J. *Through Unexplored Asia* (Dana Estes and Co., Boston, 1899).

36. Grenard, F. *Tibet–The Country & Inhabitants* (Hutchinson & Co., NY, 1904).

37. Kozloff, P. K. Through Eastern Tibet and Kam. *Geog. J.* **31**, 522–534 (1908).

38. Hedin, S. *Trans-Himalaya. Vol 1.* (Macmillan, NY, 1910).

39. Dunlop, R. H. W. *Hunting in the Himalaya* (Richard Bentley, London, 1860).

40. Hedin, S. Four years' travel in central Asia. *Geog. J.* **12**, 240–258 (1898).

41. Kennion, R. L. *Sport and Life in the Further Himalaya* (William Blackwood & Sons, Edinburgh, 1910).

42. Hedin, S. *Trans-Himalaya. Vol. III* (Macmillan, NY, 1913).

43. Rockhill, W. W. in *Hunting in Many Lands*, T. Roosevelt, G. G. Bird, Eds. (Forest and Stream, NY, 1895), pp. 255–277.

44. Littledale, S. G. A journey across Central Asia. *Geog. J.* **3**, 445–475 (1894).

45. Crosby, O. T. *Tibet and Turkestan* (G. P. Putnam's Sons, NY, 1905).

46. Fergusson, W. N. *Adventure, Sport and Travel on the Tibetan Steppes* (Charles Scribner's Sons, NY, 1911).

47. Huc, R. Travels in Tartary, Thibet. 1850 Hazlitt, Travels in Tartary, Thibet and China, 1844–1846, 2 volumes. London, Office of the National Illustrated Library, n. d. (1851), (1898).

48. Cunningham, A. *Ladak, Physical, Statistical, and Historical with Notices of the Surrounding Countries* (W. H. Allen Co., London, 1854).

49. Shaw, R. *Visits to the High Tartary, Yarkand, and Kashgar* (John Murray, London, 1871).

50. Prejevalsky, N. *From Kulija across the Tien Shan to Lob-Nor. Vol. 2* (Sampson, Low, Marston, Searle, and Rivington, London, 1876).

51. Gill, W. J. *The River of Golden Sand. The Narrative of a Journey through China and Eastern Tibet to Burmah* (John Murray, London, 1880).

52. Walker, J. T. Four years' journeyings through great Tibet. *Proc. Royal Geog. Soc. Monthly Rec. Geog., New Monthly Series* **7**, 65–92 (1885).

53. W. T. Blanford, *The Fauna of British India, Including Ceylon and Burma. Mammalia* (Taylor and Francis, London, United Kingdom, 1888)

54. Rockhill, W. W. , Through Northern China to the Koko-Nor. *Cent. Illust. Monthly Mag.* **41**, 4–17 (1890).

55. Hooker, J. D. *Himalayan Journals: Notes of a Naturalist* (Ward, Lock, Bowden and Co., London, 1891).

56. Rockhill, W. W. *Explorations in Mongolia and Tibet* (Smithsonian Institution, Government Printing Office, Washington, 1893).

57. Pratt, A. E. *To the Snows of Tibet through China* (Longmans, Green and Co., London, 1892).

58. Littledale, S. G., A journey across Tibet From North to South and West to Ladak. *Geog. J.* **5**, 453–483 (1896).

59. Ward, A. E. *The Sportsman's Guide to Kashmir and Ladak* (Calcutta Central Press, Calcutta, India, 1887).

60. Hedin, S. *Through Asia* (Harper and Brothers, NY, 1899).

61. Landor, A. H. S. *In the Forbidden Land* (William Heinemann, London, 1899).

62. Rockhill, W. W. An American in Tibet. *Century Mag*. **39**, 3–17 (1890).

63. Taylor, N. *Ibex Shooting on the Himalaya* (Sampson Low, Marston and Co., London, 1903).

64. Rawlings, C. G. Exploration of Western Tibet and Rudok. *Geog. J.* **25**, 414–429 (1905).

65. Sandberg, G. *Tibet and Thibetans* (Brighton, NY, 1906).

66. Kozloff, P. K. Through Eastern Tibet and Kam. *Geog. J*. **31**, 402–415 (1908).

67. Landor, A. H. S. *An Explorer's Adventures in Tibet* (Harper and Brothers, NY, 1910).

68. Wallace, H. F. *The Big Game of Central and Western China* (Duffield and Co., NY, 1913).

69. Ward, F. K. Wanderings of a naturalist in Tibet and Western China. *Scottish Geog. Mag*. **29**, 341–350 (1913).

70. Bailey, F. M. Exploration on the Tsangpo or Upper Brahmaputra. *Geog. J*. **44**, 341–360 (1914).

71. Wilson, E. H. *A Naturalist in Western China* (Doubleday, Page & Co., NY, 1914).

72. Ward, A. E. Game animals of Kashmir. *J. Bombay Nat. Hist. Soc.* **29**, 23–35 (1921).

73. Ward, F. K. *In Farthest Burma* (J. B. Lippincott Co., Philadelphia, 1921).

74. Hayden, H. & Cosson, C. *Sport and Travel in the Highlands of Tibet* (Hasell, Watson, and Viney, London, 1927).

75. MacDonald, D. *The Land of the Lama* (Seeley, Service, and Co., London, 1928).

76. Ward, F. K. Across Southern Tibet in 1935. *J. Royal Central Asian Soc*. **24**, 114–124 (1937).

77. Wollaston, A. F. R. The natural history of South-Western Tibet. *Geog. J*. **60**, 5–14 (1922).

78. Clutton-Brock, T.H., Albon, S. A., & Guinness, F. E.. Red deer; the ecology and behavior of two sexes. (University of Chicago Press, 1982).

79 Bowyer, R. T. Sexual segregation in ruminants: definitions, hypotheses, and implications for conservation and management. *J. Mamm*. **85**, 1039–1052 (2004).

80. Berger, J. et al., Sex differences in ecology of wild yaks at high elevation in the Kekexili Reserve, Tibetan-Qinghai Plateau, China. *J. Mamm*. **95**, 638–645 (2014).

81 ESRI. 2011a. ArcGIS Desktop Help-Cell size and resampling in analysis. Accessed May 2013.

82. Burrough, P. A. & McDonnell, R. A. Principles of Geographical Information Systems (Oxford University Press Inc., NY, 1998).

83. Modis-National http://modis.gsfc.nasa.gov/data/dataprod/dataproducts.php? MOD_NUMBER=10. Accessed December 2013.

84. http://modis.gsfc.nasa.gov/data/dataprod/dataproducts.php? MOD_NUMBER=13. Accessed December 2013.

85. Fortin, D., *et al*. Group-size-mediated habitat selection and group fusion-fission dynamics of bison under predation risk. *Ecology* **90**, 2480–2490 (2009).

86. Schaller, G. B. *Wildlife of the Tibetan Steppe* (Univ. Chicago Press, 1998).

87. Boyce, M. S., & McDonald, L. L. Relating populations to habitats using resource selection functions. *Trends Ecol. Evol*. **14**, 268–272 (1999).

88. Manly, B. F. J., McDonald, L. L., Thomas, L., McDonald, L., & Erickson, W. P. (eds.), *Resource Selection by Animals: Statistical Analysis and Design for Field studies. Second Edition.* (Kluwer, Boston, 2002).

89. Burnham, K. P., & Anderson, D. R. *Model Selection and Multimodel Inference* (Springer, NY, 2002).

90. R Core Team, R: A language and environment for statistical computing. R Foundation for Statistical Computing, Vienna, Austria. URL (http://www.R- project.org/) (2013). Accessed December 2013.

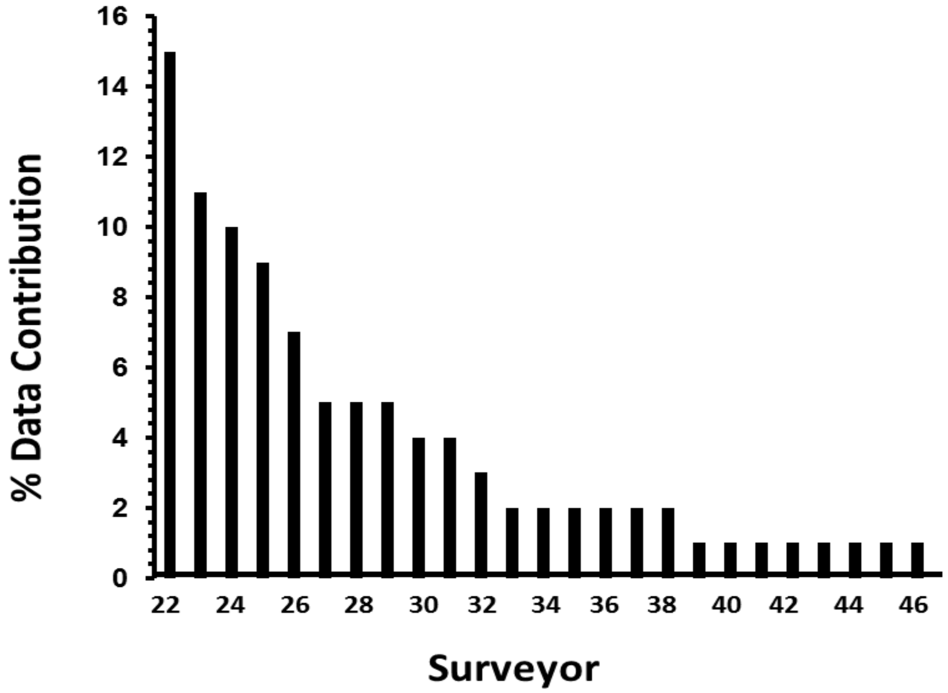

Fig. S1. Percentage of 148 data points on wild yak 2-dimensional habitat use contributed by explorers of the Tibetan-Himalayan region. Surveyor numbers in S-References.

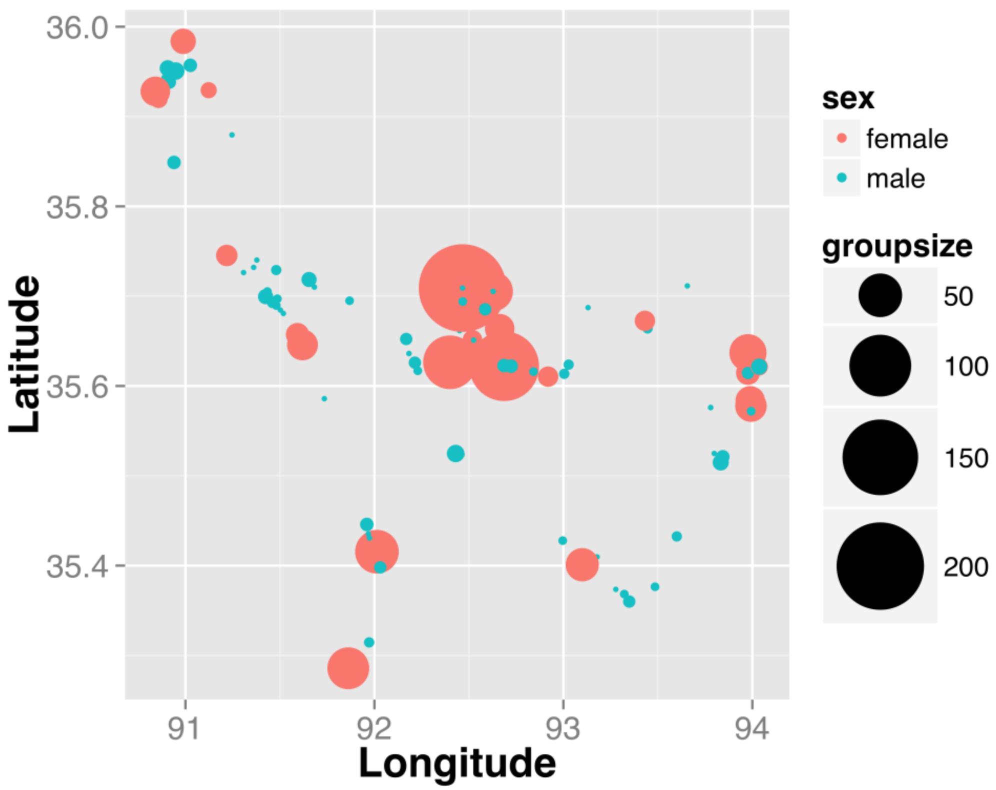

Fig. S2. Scatter plot depicts relationships between group size and sex and latitude and longitude across the Kekexili study region during 2006 and 2012.

Table. S1. Model selection results for Wild Yak resource selection functions from late fall, winter, 2006 and 2012 on the Tibetan Plateau. For each model, the structure, number of parameters K, Log-Likelihood, AICc, □AIC, AIC weight, and the cumulative AIC Weight is shown. Parameter definitions are as follows: NDVI = normalized difference vegetation index for the previous summer's growing season (July-August); Snow = percentage snow cover occurring during the survey; Slope = degree's slope of the terrain; DG = distance to nearest glacier edge in km; A_glacier = area of the nearest glacier edge in km^2; Grp = group size interactions from Eq. 1; Sex = sex-specific interactions with the same.

**Model Structure**	***K***	***LL***	**AICc**	**Δ AICc**	**AIC W_t**	**Cum W_t**
NDVI + Snow + Slope + DG+ Grp*NDVI + Snow*Grp *+ Slope*Grp	8	-414.59	845.28	0	0.82	0.82
NDVI + Snow + Slope + DG + Sex*NDVI + Sex*Snow	7	-418.94	851.96	6.68	0.03	0.84
NDVI + Snow + Slope + DG + A_glacier + Grp*Slope	7	-418.96	852.01	6.72	0.03	0.87
NDVI + Slope + DG+ A_glacier	5	-421.1	852.25	6.97	0.03	0.9
NDVI + Snow + Slope + DG + Sex*NDVI + Grp*Slope	8	-418.52	853.14	7.85	0.02	0.91
NDVI + Snow + Slope + DG + A_glacier + Sex*Snow + Grp*Slope	8	-418.53	853.17	7.89	0.02	0.93
